# Maternal health literacy on mother and child health care: A community cluster survey in two southern provinces in Laos

**DOI:** 10.1371/journal.pone.0244181

**Published:** 2021-03-29

**Authors:** Sysavanh Phommachanh, Dirk R. Essink, Pamela E. Wright, Jacqueline E. W. Broerse, Mayfong Mayxay

**Affiliations:** 1 Institute of Research and Education Development, University of Health Sciences, Ministry of Health, Vientiane, Lao PDR; 2 Vrije Universiteit Amsterdam, Athena Institute and Amsterdam Public Health Institute, Amsterdam, The Netherlands; 3 Guelph International Health Consulting, Amsterdam, The Netherlands; 4 Lao-Oxford-Mahosot Hospital-Wellcome Trust Research Unit (LOMWRU), Microbiology Laboratory, Mahosot Hospital, Vientiane, Lao PDR; 5 Centre for Tropical Medicine and Global Health, Nuffield Department of Clinical Medicine, University of Oxford, Oxford, United Kingdom; Charite Universitatsmedizin Berlin, GERMANY

## Abstract

**Rational:**

Increased maternal health literacy (MHL) has contributed considerably to maternal and child health outcomes in many countries. Malnutrition, and low coverage of child vaccination and breastfeeding are major health concerns in Laos, but there is little insight into mothers’ literacy on these issues. The aim of this study was to identify the level of MHL of Lao mothers and to explore factors influencing it, in order to provide evidence that can inform policies and planning of health services.

**Methods:**

A cross-sectional survey was conducted using a questionnaire on health literacy (ability to access, understand, appraise and apply health-related information) in relation to care during pregnancy, childbirth, and the postpartum period. We interviewed 384 mothers with children aged under five years; 197 from urban and 187 from rural areas. Descriptive and inferential statistics were applied to analyze the data.

**Results:**

Overall, MHL of Lao mothers was very low in both urban and rural areas; 80% of mothers had either inadequate or problematic MHL, while only 17.4% had sufficient and 3.5% excellent MHL. The MHL scores were significantly higher in urban than in rural areas. One third of mothers found it very difficult to access, understand, appraise and apply information on mother and child (MCH). Health personnel were the main source of MCH information for the mothers. Years of schooling, own income, health status, and number of ANC visits significantly predicted a higher level of MHL (R square = 0.250; adjusted R square = 0.240, *P =* <0.001).

**Conclusions:**

MHL of Lao mothers was inadequate in both urban and rural areas. Socio-demographics and key practices of the mothers were significantly associated with a better level of MHL. Health education on MHL to mothers in both urban and rural areas needs attention, and could best be done by improving the quality of health providers’ provision of information.

## Introduction

Maternal and child mortality remains a global concern, particularly in low- and middle-income countries [1,2]. In Laos, the maternal mortality ratio is one of the highest in Asia with 197 deaths per 100,000 live births [3]. Maternal health literacy (MHL) is the ability of mothers to access, understand, appraise and apply information on mother and child health that contributes to reducing maternal and child mortality [4–8], which subsequently contributes to achievement of sustainable development goals numbers 2 (zero hunger: reducing underweight and stunting among young children) and 3 (good health and well-being: reducing maternal and child mortality ratio) [9,10]. Previous studies in Nigeria demonstrated that there was a significant relationship between MHL and pregnancy outcomes [11] and children’s nutritional status [12]. Another study in India also showed that better MHL was independently associated with higher child vaccination, leading to the suggestion that “initiatives targeting health literacy could improve vaccination coverage” [13].

Low MHL is one of several important factors, including social and demographic issues, poor accessibility and poor quality of maternal and child health services [14–20] that contribute to poor mother and child health outcomes and to unhealthy practices of Lao mothers. For example, malnutrition continues to be a serious health concern, with approximately 33% of Lao children under five years stunted, 21.1% underweight and 9.0% wasted [14,21].

In Laos, traditional practices are still commonly followed during pregnancy and the postpartum period, for example, the practice of eating dry food with only rice and ginger as well as staying on a heated bed during the postpartum period to stop bleeding and keep the body dry and healthy. A lack of awareness about maternal and child health would probably lead to unhealthy practices such as food taboos, particularly low intake of protein-rich foods such as, meat, fish, and eggs [22]. Even in the urban setting of Vientiane, 97% of women stayed for one or two weeks on a hot bed under which burning coals are placed; and 90% of postpartum women practiced food taboos [22]. One Lao study illustrated that malnutrition was associated with maternal health knowledge and that children of the mothers with greater health knowledge had lower rates of stunting [18]. Also, child immunization coverage against preventable diseases is very low (48.3–83.1%) in Laos, with a full vaccination rate of only 40.7% [14]. Despite the availability of a comprehensive vaccination service, the population is apparently not yet sufficiently aware of the benefits of immunization and children are not immunized. A previous study demonstrated that mothers had weak knowledge about vaccine-preventable diseases and about immunization schedules associated with child vaccination [15].

Lao women could possibly access different sources of mother and child health (MCH) information, such as media (through smart phone, TV, or radio), health personnel (doctor, nurse, midwife, and other health workers), and social networks (friend, relative, and neighbor). Our recent studies demonstrated that women received MCH information from health providers when they accessed ANC services at health facilities [16,17,23]. However we do not know other sources of MCH information that women might receive, besides outreach activities of health workers providing MCH service in the villages [24]. Community campaigns by health workers with the assistance of village health volunteers and local authorities might be another important source of MCH information for women.

Lack of comprehensive awareness of MCH information in Laos is an important factor for maternal and child health outcomes as mentioned above, but we do not yet know to what extent mothers have health literacy on mother and child health care, and what factors may be influencing maternal health literacy.

The conceptual model of health literacy refers to “the personal characteristics and social resources needed for individuals and communities to access, understand, appraise and apply information and services to make decisions about health” [6]. This conceptual model would help to measure the level of maternal health literacy in relation to accessing, understanding, appraising, and applying information on mother and child health care during pregnancy and childbirth and in the postpartum period. Although health literacy can support individuals to take control of decisions and actions that will affect their health status [7], MHL remains poor in both low- and middle-income countries and high-income countries. For example, 39% of Indian mothers, 34% of mothers in Iran, 83% of rural perinatal African-American mothers, 72% of mothers in Texas (USA) and 24% of mothers in Philadelphia had scores of ‘inadequate’ when tested for MHL [13,25–28].

Factors that influence MHL are related to socio-demographic characteristics of mothers as found in previous studies and certain key practices of mothers in relation to pregnancy, childbirth, and postpartum care, as taken from WHO guidelines [29]. A study in Iran showed a significant relationship between MHL and both women’s education and family income [24]. Another study in Nigeria demonstrated that there was a significant positive correlation between ANC visits and MHL [11], and in Texas (USA), MHL was positively correlated with education, income, language, social support, parenting self-efficacy, and early parenting practices, but negatively correlated with number of children [28].

There has been little research on health literacy in Laos. One study in Vientiane city measured general health literacy among university students [30] while another focused on sexual and reproductive health literacy among school adolescents [31]. Both studies indicated low levels of health literacy in those parts of the population. To the best of our knowledge, no studies have yet been conducted on MHL of Lao mothers.

The primary objective of this study was to determine the levels of maternal health literacy on topics of mother and child health care in urban and rural areas, and to determine the factors influencing MHL among Lao mothers. Gaining insights into MHL contributes to raising awareness on MHL. The factors associated with MHL could enable policy makers to develop more targeted interventions.

## Methods

### Study design and site

A cross-sectional study was conducted from April to July 2018 at the community level in the two southern provinces of Saravane and Attapeu. Two of the eight districts (one urban and one rural) and two of the five districts (one urban and one rural) of Saravane and Attapeu provinces, respectively, were purposively selected. Four villages (two urban and two rural) of two selected districts in each province were chosen for the study (see below in the sampling technique). This study was part of a larger research project on maternal and child health care, which started in 2017 in these two provinces with a special emphasis on quality improvement of ANC [22,32].

### Sample size and sampling method

Sample size was calculated using a formula (http://www.calculator.net/sample-size-calculator.html) based on the total number of mothers with children aged under five years according to the latest available data from 2016 (192,000) [14], using a 95% confidence interval and margin of error of 5%. Since there was no information on MHL available in Laos, we assumed a proportion of eligible mothers with adequate MHL level of 50%. Therefore it was estimated that we had to recruit at least 384 mothers for this study. Given that 5% of eligible mothers may not be available and/or willing to participate, an additional 19 mothers should be invited for an interview–therefore a total sample size of 403 mothers was considered adequate for the study.

The provinces and districts were purposively selected because of their involvement in a larger study looking into different aspects of mother and child health. A two-stage cluster sampling was done at the levels of village and household as described below.

- **Stage 1 village selection (cluster sampling):** A list was made of all villages (clusters) in each district. Four villages (four clusters) were selected from districts using a simple random sampling method, generated by Microsoft Excel, giving a total of eight villages (8 clusters) for the two districts of the two provinces.
- **Stage 2 household selection:** A list of all households of mothers with children aged under five years was made for each village (cluster) just before the survey. The Probability Proportional to Size (PPS) sampling method was applied. A village (cluster) with more eligible households had a greater number of households to be studied, and the total sample size of 403 households was allocated to each sample village/cluster. Then the number of the households to be studied in each village/cluster was selected using a simple random sampling method, also generated by Microsoft Excel.

### Population and recruitment

Eligible participants were Lao women living in the selected areas with children aged under five years. Since child immunization is one important component of MCH and we wanted to measure literacy on that topic as one of the MHL items, we therefore aimed to include mothers with children whose ages were within the period of receiving basic vaccination (from birth to 23 months). However, in the area of the study, to reach the desired sample size we had to select mothers whose children were aged from birth to 5 years. This is also consistent with most child health policies in Laos, aimed at children under 5 years. We acknowledge that there could be recall bias when we enrolled the mothers with older children. However, the mothers were only asked to reflect on the information related to the last child, not any earlier children.

The study unit was a household, and one eligible mother per household was included in the study. Each head of the village assisted to inform the household members two days before the planned interviews. If interviewers found respondents who were not eligible or declined to participate, then the interviewers randomly selected another household to get a new respondent until sufficient data was collected. The response rate was 95%, so finally 384 mothers were enrolled. The reasons for the 19 mothers not to participate were: six no longer lived in their villages and 13 were not available at the time planned for the interview. No mothers declined to be interviewed.

### Research tools

The health literacy model of Sørensen is widely used in Europe and Asia [28–31,33]. There was no specific tool for MHL in Laos, although it has been measured in other countries [7,34–36]. The research tool used in this study was based on the one previously used for general literacy tool (rating ability to access, understand, appraise/judge/evaluate, and apply/use health information), adapted to MCH care. Maternal health literacy refers to the ability of mothers to access, understand, appraise, and apply information on mother and child health care during pregnancy, at childbirth and in the postpartum period with emphasis on specific attention was paid to key practices of ANC visits, childbirth, and postpartum care at health facilities, nutrition, and immunization, following WHO guidelines and the National Strategic Action Plan [24,29,37].

The research tool for data collection covered three sections:

1. socio-demographic data such as age, religion, ethnicity, language, literacy, education, and income, taken from previous studies [4,11,28,33,38];
2. possibly related factors or practices (positive and negative), such as ANC visits, place of childbirth, number of children, food restrictions/taboos, the use of hotbeds, smoking, and receiving information, all taken from previous studies [22,39,40]; and
3. MHL in relation to the ability of mothers to access, understand, appraise, and apply information on mother and child health care during pregnancy, at childbirth and in the postpartum period, according to WHO guidelines and the National Strategic Action Plan [24,29] as mentioned above.

In sections 1 and 2, literacy was defined as the ability of mothers to read, and education was recorded as the number of years of schooling. Ages of mothers, own income per month, and years of schooling were categorized into two groups by median (range). Mothers were asked to rate their own health status from 0 to 10; their status was categorized as poor if their score fell below the mean and good if the score was at the mean or higher. Complete child vaccination means that a child has completed all basic required vaccinations from childbirth to the age it had reached at the time of survey (can be up to 23 months), based on the Expanded Program Immunization schedule of Laos; the vaccination card was checked to confirm the completeness. Food restrictions/taboos mean that mothers followed local traditional food restrictions such as “low intake of protein-rich foods such as meat, fish, and eggs” during pregnancy and the postpartum period. Hotbed stay refers to the postpartum mother staying in a bed under which hot coals are placed and maintained for one or two weeks. Examples of the questions asked are: *“Did you visit ANC for the last child*? *Did you have food restriction/taboo for the last childbirth*? *Did you practice a hotbed stay for the last childbirth*? *Have you ever heard any information on mother and child health care*? *If yes…where have you ever received/heard information on mother and child health care*?*” How is your health in general*? *Please ask the respondent to grade her health condition using a ruler with 1 to 10 scores*.*”*

In section 3, the MHL questionnaire comprised 49 items in relation to mother and child health care during pregnancy, childbirth and postpartum period, which were developed based on WHO guidelines and the National Strategic Action Plan [24,29]. Items of MHL were adapted to the structure of a health literacy survey tool in Asia [41] and sexual reproductive health literacy tool in Laos [31]. It included 15 items on accessing information about prenatal care, childbirth, and postpartum care, 11 on understanding information about nutrition and immunization, 12 on appraising information about maternal and child health care, and 11 on applying information about maternal and child health care practices. Examples of questions are: “*how is it easy for you to find health education materials on MCH (e*.*g*. *brochure*, *poster*, *etc) disseminated in the village when you want to know*? …*to understand information that If a pregnant woman practiced food restriction during pregnancy*, *it will be a risk of low birth weight and unhealthy baby*?….*to judge whether the information you received from health providers is sufficient*? …*to use ANC service earlier when recognize that you have missed period to make sure on getting pregnancy”*.

### Measurement of health literacy

To measure MHL, each item was rated on a 4-point Likert scale (4 very difficult, 3 difficult, 2 easy, and 1 very easy), with a possible lowest mean score of 1 and a possible highest mean score of 4. The mean of all participating items for each individual was calculated for their specific index score. The indices for health literacy were standardized to unified metrics from 0 to 50 using the formula index = (mean-1)*50/3). A minimum value of the index score was 0 if minimal possible value of the mean was 1 and 50 was maximum value of index score [36,41,42]. The cut-off based on health literacy measurements in Asia and in Europe [36,41,42]; levels were reported as ‘inadequate’ (0–25), ‘problematic’ (>25–33), ‘sufficient’ (>33–42) and ‘excellent’ (>42–50) levels of health literacy. For certain groups of final results, the ‘sufficient’ and ‘excellent’ levels were combined to a single level, called ‘adequate health literacy’ index scores (33–50) and below 33, index scores were combined to become ‘inadequate’. These two levels facilitate comparison of mothers’ socio-demographics and key practices with adequate and inadequate maternal health literacy. The total index scores were applied for correlation analysis (using either Pearson or Spearman tests), and multiple-linear regression helped to predict maternal health literacy by independent variables.

The research tools were firstly developed in English; thereafter back and forth translation was made between English and Lao languages to ensure accuracy. The Lao language was used in the field work; it is the language mainly used in this area. The research assistant did the translation from English to Lao language, then the PI translated back from Lao to English. The local supervisor helped with a final edit before the pilot test. The Lao language version was pilot tested at villages in the study area different from the study sites but with similar conditions, with 30 mothers who had children under five years old for internal consistency with Cronbach’s alpha, for which a value of greater than or equal to 0.7 indicates satisfactory reliability [33]; the consistency value for this study was 0.934.

### Data collection

Eight research assistants were recruited and trained for data collection. Authors SP and MM trained them for four days on how to do face-to-face interviews and on completing the questionnaires accurately. During the training, the eight data collectors practiced data collection in communities not included in the study. We conducted interviews in the participants’ households, for the privacy and convenience of the respondents. During the interview the information was recorded in the answer sheet and the interviewer checked the mother’s record book and vaccination card; child complete vaccination, ANC visits, and place of childbirth (if that was at a health facility) could be checked against the mothers’ responses. Nearly all (95%). Before interviews, mothers were fully informed about the study objectives and given an overview of the interview process. They were assured of anonymity to ensure confidentiality, and were told that they could refuse to answer any specific question or to withdraw from the interview at any time without explaining why. The completion of each answer sheet was double checked by the research team and team leaders to ensure the quality and completeness of the data. The approximate length of each interview was 45–60 minutes.

### Data analysis

Data were entered into Microsoft Excel (2011) for Mac then transferred to ‘IBM SPSS 25’ for analysis (IBM Corp., Armonk, NY). Descriptive statistics, such as percentage, mean (standard deviation; SD) and median (range), were applied to describe personal information and the patterns of maternal health literacy.

Comparison of proportions between two groups was made using χ^2^ or Fisher’s exact tests as appropriate. Mann Whitney U test or Student’s t tests were applied to compare medians or means between groups (see Tables 1 and 4). In multiple logistic regressions, socio-demographic and key practices of Lao mothers were identified as independent variables and dependent variables as inadequate and adequate MHL levels. These variables were entered forwardly into the model to determine the Odds Ratio (OR), 95% confidence interval (95% CI), and significance level when P-Value was less than 0.05 (see Table 5).

**Table 1:** Characteristics and key practices of the study mothers^a^.

Variable	Total N = 384 (%)	Urban n (%)	Rural n (%)	P-Value
**Age (Years)**				
<28	184 (47.9)	73 (37.1)	111 (59.4)	
> = 28	200 (52.1)	124 (62.9)	76 (40.6)	<0.001^(Z)^
Median (Range)	28 (14–46)	30 (14–46)	25 (14–45)	<0.001^(M)^
**Religion**				
1 –Buddhist	309 (80.5)	191 (97.0)	118 (63.1)	<0.001^(Z)^
2 –Others	75 (19.5)	6 (3.0)	69 (36.9)	
**Mother tongue/native language**				
1 –Lao	272 (70.8)	189 (95.9)	83 (44.4)	<0.001^(Z)^
2 –Dialects	112 (29.2)	8 (4.1)	104 (55.6)	
**Ethnic group**				
1 –Lao Lum	265 (69.0)	189 (95.9)	76 (40.6)	
2 –Others	119 (31.0)	8 (4.1)	111 (59.4)	<0.001^(Z)^
**Literacy**				
Able to read	293 (76.3)	165 (83.8)	128 (68.4)	
Unable to read	91 (23.7)	32 (16.2)	59 (31.6)	<0.001^(Z)^
Education (years of schooling)				
Median (range) years of schooling	7 (0–19)	11 (0–19)	5 (0–15)	<0.001^(M)^
**Marital status**				
1 –Married	358 (93.2)	184 (93.4)	174 (93.0)	0.891^(Z)^
2 –Others (single, separated, divorced, widower)	26 (6.8)	13 (6.6)	13 (7.0)	
**Own income per month (LAK) ^b^**				
> 400,000	187 (48.7)	127 (64.5)	60 (32.1)	
< = 400,000	197 (51.3)	70 (35.5)	127 (67.9)	<0.001^(Z)^
Median (range)	400,000 (0–8,000,000)	800,000 (0–8,000,000)	200,000 (0–8,000,000)	<0.001^(M)^
**Grading of overall health status**				
Good	243 (63.3)	139 (70.6)	104 (55.6)	0.002
Poor	141 (36.7)	58 (29.4)	83 (44.4)	
Mean (SD) over 10	7.8 (1.8)	8 (1.6)	7.51 (1.9)	0.003^(T)^
**Number of ANC visits**				
4–9	151 (39.3)	83 (42.1)	68 (36.4)	0.247^(Z)^
0–3	233 (60.7)	114 (57.9)	119 (63.6)	
Median (range)	3 (0–9)	3 (0–9)	3 (0–9)	0.317^(M)^
**Food restriction practice during pregnancy**				
Yes	87 (22.7)	20 (10.2)	67 (35.8)	<0.001^(Z)^
No	297 (77.3)	177 (89.8)	120 (64.2)	
**Place of birth for the last child**				
Health facilities	281 (73.2)	165 (83.8)	116 (62.0)	<0.001^(Z)^
Others	103 (26.8)	32 (16.2)	71 (38.0)	
**Hotbed stay after delivery for the last childbirth**				
Yes	345 (89.8)	178 (90.4)	167 (89.3)	0.733^(Z)^
No	39 (10.2)	19 (9.6)	20 (10.7)	
**Food restriction practice postpartum**				
Yes	278 (72.4)	126 (64.0)	152 (81.3)	<0.001^(Z)^
No	106 (27.6)	71 (36.0)	35 (18.7)	
**Child completed vaccination**				
Yes	166 (43.2)	101 (51.3)	65 (34.8)	0.001^(Z)^
No	218 (56.8)	96 (48.7)	122 (65.2)	
**Currently smoking**				
Yes	99 (25.8)	28 (14.2)	71 (38.0)	
No	285 (74.2)	165 (85.8)	116 (62.0)	<0.001^(Z)^
**Currently drinking any kind of alcohol**				
Yes	272 (70.8)	149 (75.6)	123 (65.8)	
No	112 (29.2)	48 (24.4)	64 (34.2)	0.034^(Z)^

(a) Data shown as number (%) unless indicated.

(b)1 USD = 8,630 LAK.

(Z) Z test.

(T) T-student test.

(M) Mann-Whitney U Test.

### Ethical approval and participants’ consent

This study was part of a research project on the situation analysis of MCH care in Laos, which received ethical approval from the Ethics Committee of the University of Health Sciences, Ministry of Health, Laos. We obtained written consent from eligible mothers before beginning each interview. Fingerprints were provided by mothers who could not sign to give consent.

## Results

### Characteristics and key practices of the study mothers

A total of 384 mothers, 197 from urban and 187 from rural areas, were enrolled in the study. Their characteristics and practices are shown in Table 1. The overall median (range) age was 28 (14–46) years. Most of the mothers could read and speak the Lao language (70.8% and 76.3%); these proportions were significantly higher in urban than in rural areas (*P*<0.001)^Z^.

The median (range) number of ANC visits made by the mothers was 3 (0–9); only one third of mothers visited ANC more than four times during their pregnancies. Two thirds of mothers had given birth at health facilities for their last child, this was also significantly higher in urban than in rural areas (*P =* <0.001^)Z^. Approximately three quarters of the mothers had practiced food restriction during the postpartum period, and 22% had practiced it during pregnancy. Nearly 90% of mothers had stayed on a hotbed in both urban and rural areas. The overall proportion of the children with complete immunization was lower than 50%, but significantly lower in rural than in urban areas (*P =* 0.001)^Z^.

### Information received by respondents on maternal and child health

Table 2 illustrates whether and how mothers had received (MCH information. Nearly 90% of mothers reported that they had received MCH information. Of these, 80% said they received it from health personnel at health facilities and 58% from health personnel during outreach sessions. Other sources of MCH information included media and their social network (family, relatives, friends, and neighbors) but these were reported by less than 50% of the respondents as a source of information.

**Table 2:** Receiving information on maternal and child health^a^.

Variables	Total	
	(N = 384)^b^	(%)
**Had mothers ever heard any MCH information?**		
Yes	341	88.8
No	43	11.2
**Sources of MCH information**		
1. Media (TV, radio, and or smart phone)	174	45.3
2. Health personnel at health facilities (Doctor, nurse, midwife, and or other health workers)	306	79.7
3. Health personnel campaign/at community level (Doctor, nurse, midwife, and or other health workers together with village heath volunteer and local authority)	222	57.8
4. Social network (family, relatives, friends, and/or neighbors)	186	48.4

(a) Data shown as number (%) unless indicated.

(b) Multiple answers from mothers who had ever heard any MCH information from each source.

### Comparison of maternal health literacy levels and index scores between urban and rural mothers

Despite the mothers having received information, overall MHL levels were largely inadequate, as shown in Table 3. Approximately 80% of the mothers had either inadequate or problematic MHL, while only 17.4% reached adequate and 3.5% had excellent MHL. Two thirds of mothers found it difficult to access, understand, appraise and apply MCH information. For example, 69% of mothers reported that they encountered difficulties in finding health education materials on MCH (e.g. brochures and posters disseminated in the village) when they wanted to know more; 74% of mothers found it difficult to understand information, for example, that after delivery, the mother should eat fruits. Also, more than two thirds of mothers had trouble appraising and applying any MCH information they received. For example, 77.9% of mothers reported that they found it very difficult to judge whether information about the benefits of food diversity for mother and child was correct or not; 84.4% of mothers reported that they could not easily use information about physical and mental health care (e.g. exercise, swimming, yoga, listening to music, getting enough sleep, keeping a good mood) during pregnancy. The total mean MHL scores varied between urban and rural areas, although the index scores were very low in both (Table 3). The overall MHL index score was 30, but it was significantly higher in urban than in rural areas (*P =* 0.001)^M^.

**Table 3:** Comparison of MHL levels and index scores between urban and rural mothers ^a^.

MHL level	Total N = 384 (%)	Urban n (%)	Rural n (%)	P-value
**Access to information**				
Inadequate (0–25)	161 (41.9)	73 (37.1)	88 (47.1)	
Problematic (>25–33)	170 (44.3)	91 (46.2)	79 (42.2)	
Sufficient (>33–42)	30 (7.8)	18 (9.1)	12 (6.4)	0.150^(Z)^
Excellent (>42–50)	23 (6.0)	15 (7.6)	8 (4.3)	
Index score 50 max: Median (range)	28 (0–50)	32 (0–50)	26 (0–50)	0.002^(M)^
**Understanding information**				
Inadequate (0–25)	127 (33.1)	58 (29.4)	69 (36.9)	
Problematic (>25–33)	149 (38.8)	65 (33.0)	84 (44.9)	
Sufficient (>33–42)	67 (17.4)	45 (22.8)	22 (11.8)	<0.001^(Z)^
Excellent (>42–50)	41 (10.7)	29 (14.7)	12 (6.4)	
Index score 50 max: Median (range)	32 (0–50)	33 (0–50)	30 (0–50)	<0.001^(M)^
**Appraising information**				
Inadequate (0–25)	123 (32.0)	55 (27.9)	68 (36.4)	
Problematic (>25–33)	181 (47.1)	91 (46.2)	90 (48.1)	
Sufficient (>33–42)	59 (15.4)	38 (19.3)	21 (11.2)	0.065^(Z)^
Excellent (>42–50)	21 (5.5)	13 (6.6)	8 (4.3)	
Index score 50 max: Median (range)	31 (0–50)	31 (0–50)	29 (0–50)	0.004^(M)^
**Applying information**				
Inadequate (0–25)	131 (34.1)	57 (28.9)	74 (39.6)	
Problematic (>25–33)	181 (47.1)	95 (48.2)	86 (46.0)	
Sufficient (>33–42)	45 (11.7)	30 (15.2)	15 (8.0)	0.052^(Z)^
Excellent (>42–50)	27 (7.0)	15 (7.6)	12 (6.4)	
Index score 50 max: Median (range)	30 (0–50)	30 (0–50)	27 (0–50)	0.002^(M)^
**Grand total index scores (50 max)^b^ 30 (0–50)**		**32 (0–50)**	**27 (0–50)**	**0.001**^**(M)**^

(a) Data shown as number (%) unless indicated.

(b) Possible lowest index score is 0 and highest index score is 50.

(z) Z test.

(M) Mann-Whitney U test.

### Comparison between mothers with inadequate and adequate maternal health literacy levels

Socio-demographics and key practices of Lao mothers who had inadequate and adequate MHL levels are shown in Table 4. Significantly higher proportions of appropriate MHL levels were found among mothers who speak the Lao language, who have high income, who frequently visited ANC, who gave birth at health facilities, and who had a child with complete vaccination, as compared to the other group of mothers (*P =* 0.001^Z^, *P =* <0.001^ZM^, P = 0.011^Z^, *P = 0*.*003*^*Z*^, *P =* 0.006^Z^, *P =* 0.002^Z^, and *P =* 0.042^Z^ respectively). However, having heard MCH information was not significantly associated with MHL.

**Table 4:** Comparison of socio-demographic features between mothers with inadequate and adequate MHL levels^a^.

Variable	Total N = 384 (%)	Health literacy level^b^		P-Value
		Inadequate n (%)	Adequate n (%)	
**Age**				
<28 years	184 (49.9)	152 (50.5)	32 (38.6)	
> = 28 years	200 (52.1)	149 (49.5)	51 (61.4)	0.054^(Z)^
**Religion of mothers**				
1 –Buddhist	309 (80.5)	231 (76.7)	78 (94.0)	<0.001^(Z)^
2 –Other religious	75 (19.5)	70 (23.3)	5 (6.0)	
**Mother tongue/native language of mothers**				
1 –Lao	272 (70.8)	201 (66.8)	71 (85.5)	0.001^(Z)^
2 –Dialects	112 (29.2)	100 (33.2)	12 (14.5)	
**Ethnic group of mothers**				
1 –Lao Lum	265 (69.0)	198 (65.8)	67 (80.7)	0.009^(Z)^
2 –Other minority ethnic group	119 (31.0)	103 (34.2)	16 (19.3)	
**Education level**				
Able to read	293 (76.3)	226 (75.1)	67 (80.7)	0.285^(Z)^
Unable to read	91 (23.7)	75 (24.9)	16 (19.3)	
**Marital status**				
1 –Married	358 (93.2)	278 (92.4)	80 (96.4)	0.146^(Z)^
2 –Others	26 (6.8)	23 (7.6)	3 (3.6)	
**Own income per month (LAK)^c^;**				
< = 400,000	197 (51.3)	171 (56.8)	26 (31.3)	
>400,000	187 (48.7)	130 (43.2)	57 (68.7)	<0.001^(Z)^
Median (range)	400,000 (0–800,000)	300,000 (0–800,000)	100,000 (0–500,000)	<0.001^(M)^
**Grading of health in general**				
Good	243 (63.3)	183 (60.8)	60 (72.3)	0.054^(Z)^
Poor	141 (36.7)	118 (39.2)	23 (27.7)	
**Ever heard any MCH information**				
Yes	341 (88.8)	263 (87.4)	78 (94.0)	0.091^(Z)^
No	43 (11.2)	38 (12.6)	6 (6.0)	
**Number of ANC visits**				
4–9	151 (39.3)	106 (35.2)	45 (54.2)	0.006^(Z)^
1–3	180 (46.9)	149 (49.5)	31 (37.3)	
0	53 (13.8)	46 (15.3)	7 (8.4)	
**Food restriction practice during pregnancy**				
Yes	87 (22.7)	71 (23.6)	16 (19.3)	0.406^(Z)^
No	297 (77.3)	230 (76.4)	67 (80.7)	
**Place of birth of the last child**				
At health facilities	281 (73.2)	209 (69.4)	72 (86.7)	0.002^(Z)^
At home	103 (26.8)	92 (30.6)	11 (13.3)	
**Hotbed stay after delivery for the last childbirth**				
Yes	345 (89.8)	269 (89.4)	76 (91.6)	0.575^(Z)^
No	39 (10.2)	32 (106)	7 (8.4)	
**Food restriction practice during postpartum period**				
Yes	278 (72.4)	220 (73.1)	58 (69.9)	0.562^(Z)^
No	106 (27.6)	81 (26.9)	25 (30.1)	
**Last child completed vaccination**				
Yes	166 (43.2)	122 (40.5)	44 (53.0)	0.042^(Z)^
No	218 (56.8)	179 (59.5)	39 (47.0)	
**Currently smoking**				
Yes	99 (25.8)	87 (28.9)	12 `(14.9)	
No	285 (74.2)	214 (71.1)	71 (85.5)	0.008^(Z)^
**Currently drinking any kind of alcohol**				
Yes	272 (70.8)	207 (68.8)	65 (78.3)	
No	112 (29.2)	94 (31.2)	18 (21.7)	0.90^(Z)^

(a) Data shown as number (%) unless indicated.

(b) Inadequate = inadequate + problematic; adequate = sufficient + excellent.

(c) 1 USD = 8,630 LAK.

(Z) Z test.

(M) Mann-Whitney U Test.

### Socio-demographic and key practice factors associated with MHL of Lao mothers

Factors associated with MHL level are shown in Table 5. There are three independent variables associated with the MHL level in multiple logistic regression analysis. For example, mothers who have high income, who frequently visited ANC, and who live in urban areas were significant associated with higher MHL level [*Adjusted OR =* 0.52, 95% CI (0.289–0.934, *P* = 0.029); *Adjusted OR =* 2.198, 95% CI (1.278–3.782), *P* = 0.004; and *Adjusted OR =* 0.189, 95% CI (0.268–2.582, *P* = 0.029)] respectively. However, having heard any MCH information (*Adjusted OR* = 2.631) was not significantly associated with MHL (*P* = 0.056).

**Table 5:** Socio-demographic and key practices of Lao mothers associated with MHL.

Variable	OR ^d^		95% CI ^e^		P-Value ^f^
	Crude OR	Adjusted OR	Lower	Upper	
**Age group**					
<28	1.62	0.82	0.458	1.467	0.234
> = 28	1				
**Religion of mothers**					
Buddhist	0.212	3.298	0.988	11.013	0.052
Other religious	1				
Lao	0.34	3.273	0.767	13.966	0.109
Dialects	1				
Lao	0.459	0.279	0.076	1.025	0.054
Minority ethnics	1				
**Education**					
Ability to read	0.72	0.832	0.417	1.659	0.601
Unable to read	1				
**Marital status**					
Married	0.453	1.595	0.434	5.859	0.442
Others	1				
>400,000	2.884	0.52	0.289	0.934	0.029
< = 400,000	1				
Good	0.594	1.485	0.801	2.754	0.209
Poor	1				
Yes	0.334	2.631	0.975	7.005	0.056
No	1				
Media	1.104	0.361	0.036	3.604	0.385
Others	1				
4–9	0.459	2.198	0.278	3.782	0.004
0–3	1				
At health facilities	0.347	1.763	0.819	3.759	0.147
Others	1				
Yes	0.604	1.026	0.575	1.832	0.931
No					
Urban	0.59	0.189	0.268	2.582	0.002
Rural	1				

(a) Data shown as number (%) unless indicated.

(b) Inadequate = inadequate + problematic; adequate = sufficient + excellent.

(c) 1 USD = 8,630 LAK.

(d) OR = Adjust OR.

(e) 95% CI (confidence interval) with lower and upper bound.

(f) Significant level = <0.005.

## Discussion

This is the first study conducted in Laos to measure maternal health literacy (MHL) of mothers, emphasizing the care during pregnancy, childbirth, and postpartum periods. The results show that their MHL was very poor with ~80% having either inadequate or problematic MHL. The finding also revealed that MHL was significantly lower in rural than in urban areas (*P =* 0.001), probably because urban mothers had better literacy and more years of schooling than did rural mothers, and we found that mothers’ years of schooling was significantly associated with MHL. Improving MHL in both rural and urban areas could increase mothers’ ability to make decisions and take actions that would be good for their own and their children’s health [7]. Poor MHL of the mothers has also been reported in India, in USA, and in Iran [13,25–27,43–45].

Women received mother and child health (MCH) information from different sources. A study in Vietnam indicated that family members were very important for mothers in ethnic minority communities, among other sources of MCH information (social networks, health literacy mediators, and health professionals) [4], but in our study, health workers were the main source. Most women (about 80%) had received information on MCH from health providers, either at the health facility or during health campaigns in their village, while less than half had received MCH information from media (smart phone, TV and or radio) and social networks (relative, friend, and or neighbor). We did not separately assess for different sources of information, but most of the information came from health workers, and the mothers reported having trouble appraising and applying all MCH information they received, regardless of the source.

However, getting information does not necessarily lead to improved MHL. It appears that health providers are able to reach the target group, but their communication of health messages may not be very effective. Our previous studies on ANC focusing on information and education demonstrated that very little health information was actually provided by health workers to the pregnant women. Health workers often neglected giving MCH information and when they did, their communication skills were poor. Another issue was that MCH information materials are not well developed in Laos and were seldom available or used during ANC visits [22,23,32]. When we combine the reported poor communication performance of the health workers and the lack of health literacy among the mothers, we believe that strategies and activities to strengthen health workers’ communication skills could help to improve mothers’ health literacy.

Different results have been reported in other contexts. For example, in Iran, health education of mothers was significantly associated with MHL [24] and in Texas (USA), education, family/household income, and English language were significantly associated with better MHL [25], while in our case it was the mother’s own income that was significantly positively correlated with greater MHL. A study in Mali demonstrated that own income of mothers was associated with child nutrition status [38].

In addition, our results suggest that making four or more ANC visits was significantly associated with adequate MHL. This is consistent with similar results in Nigeria [11]. Although we cannot make inferences about causal relations and/or directions, these associations do suggest that increased literacy is significantly related to positive practices. Most notably visits to ANC and immunization completion were positively correlated with increased health literacy. However, other practices, such as hotbed stay, food restrictions, and drinking alcohol were not related with maternal health literacy level, although they do pose serious health risks. Hotbed stays have been significantly associated with newborns’ skin infection and even septicemia [46]. Food taboos/restrictions during the postpartum period have been associated with infantile beri-beri (vitamin B1 deficiency) and death [47–51]. These practices are probably hard to change despite high literacy scores, because hotbed stay and traditional food restrictions are known to be deeply embedded in Lao culture [22].

### Strengths and limitations

The strengths of this study are the large sample size representing both urban and rural mothers, which could be sufficient to identify significant differences, strengthened by the sampling method, using cluster sampling at the village level, and proportional to size for the household level.

However, the study also had limitations. Cross-sectional data cannot provide causal inferences as longitudinal studies might. Furthermore, not all potentially important influencing factors were included, for example, we did not investigate social support, parenting self-efficacy, and early parenting practices [25]. Food restriction was considered a negative factor here, but in other settings the same practice may mean low intake of unhealthy foods such as sugar, white bread, processed meat, and considered a positive factor for fetal development [52]. We did not look at food restriction in detail, but another study revealed that women avoided eating vegetables, fruits, and some types of meat, as well as sauces, sugar and spices [22]. Because we did not use information on the age of the child, we do not know how long ago the birth took place and therefore cannot say whether recall bias might be less among mothers who gave birth more recently. Information on ANC visits, place of delivery, and child immunization was checked on the appropriate record cards, but we could not check in the same way for information received about breastfeeding, so there could be recall bias for that topic among mothers whose children were older.

The findings of this study reflect the Lao context, where the health personnel seem to be the main source of information for mothers, the deficiencies in providing maternal and child health information identified in earlier studies may explain why mothers still had low levels of maternal health literacy. If mothers’ knowledge and practice are to improve, the quality of information and communication skills is as important as the fact of providing information. Therefore, the quality of delivering comprehensive information by health care providers has to be improved as well.

Although the study may not be representative of the whole country, we think that the sample can be representative of at least the population in Southern Laos, because the two provinces of our study site had been randomly selected from the five provinces in the southern region in previous studies [16,17]. Nevertheless, we do have the impression that in many other areas of Laos similar results will be found. Women largely utilize similar public health services, staffed by providers that were educated in one of the few training facilities available. Also women largely utilize similar media. However, social networks and customs, which are critical for health literacy, may differ between regions.

## Conclusion

Overall, the maternal health literacy level of Lao mothers found in this study was very poor in both urban and rural areas. Health personnel were reported to be the main source of MCH information but were apparently not very effective in that aspect of their work. Socio-demographic characteristics and key practices of mothers were significantly associated with levels of MHL. To improve the level of MHL in Laos, developing better information, education and communication materials and strengthening communication skills of health workers are strongly recommended.

## List of abbreviations

ANC: Antenatal Care
IEC: Information Education Communication
MHL: Maternal Health Literacy
MCH: Maternal and Child Health

## References

[1] Zureick-Brown S, Newby H, Chou D, Mizoguchi N, Say L, Suzuki E, et al. Understanding global trends in maternal mortality. Int Perspect Sex Reprod Health. 2013;39(1):32–41. 10.1363/3903213 23584466 PMC3886625

[2] UN IGME (United Nations Inter-agency Group for Child Mortality Estimation). Levels & Trends in Child Mortality. Vol. 147, New York. 2017.

[3] WHO. Trends in Maternal Mortality: 1990 to 2015 Estimates. Vol. 16, WHO Library Cataloguing-in-Publication Data. 2015.

[4] McKinn S, Linh DT, Foster K, McCaffery K. Distributed Health Literacy in the Maternal Health Context in Vietnam. HLRP Heal Lit Res Pract. 2019;3(1):e31–42. 10.3928/24748307-20190102-01 31294305 PMC6608917

[5] DeWalt DA, Hink A. Health Literacy and Child Health Outcomes: A Systematic Review of the Literature. Pediatrics. 2009;124(Supplement 3):S265–74. 10.1542/peds.2009-1162B 19861480

[6] Dodson S, Good S, Osborne R. Health literacy toolkit for low- and middle-income countries. A series of information sheets to empower communities and strengthen health systems [Internet]. National Network of Libraries of Medicine Southeastern/Atlantic Region. 2014. Available from: http://apps.searo.who.int/PDS_DOCS/B5148.pdf?ua=1.

[7] Smith SA, Carroll LN. Data-driven maternal health literacy promotion and a postscript on its implications. 2017;37:235–52. 28972515

[8] Azugbene E. University of Georgia, Athens, GA U. Maternal Health Literacy and Maternals and Child Health Outcomes: A Review of the Literature. Ann Glob Heal. 2017;83(1).

[9] LCCO. Comments of Lao CSOs on the Lao PDR ‘ s Voluntary National Review (VNR) 2018 for Sustainable Development Goals (SDG). 2018;2–5.

[10] People L, Republic D, Partners I, People L, July DR. Lao People’s Democratic Republic: Voluntary National Review on the Implementation of the 2030 Agenda for Sustainable Development. 2018.

[11] Mojoyinola J. Influence of Maternal Health Literacy on Healthy Pregnancy and Pregnancy Outcomes of Women Attending Public Hospitals in Ibadan, Oyo State, Nigeria. African Res Rev. 2011;5(3):28–39.

[12] Sufiyan M, Umar A, Bashir S. Effect of maternal literacy on nutritional status of children under 5 years of age in the Babban-Dodo community Zaria city, Northwest Nigeria. Ann Niger Med. 2013;6(2):61.

[13] Johri M, Subramanian S V, Sylvestre M, Dudeja S, Chandra D, Koné GK, et al. Child health Association between maternal health literacy and child vaccination in India: a cross-sectional study. 2015;849–57. 10.1136/jech-2014-205436 PMC4552929 25827469

[14] Kamiya Y. Socioeconomic determinants of nutritional status of children in Lao PDR: Effects of household and community factors. J Heal Popul Nutr. 2011;29(4):339–48. 10.3329/jhpn.v29i4.8449 PMC3190364 21957672

[15] Maekawa M, Douangmala S, Sakisaka K, Takahashi K, Phathammavong O, Xeuatvongsa A, et al. Factors affecting routine immunization coverage among children aged 12–59 months in Lao PDR after regional polio eradication in Western Pacific Region. Biosci Trends. 2007;1(1):43–51. 20103866

[16] Phommachanh S, Essink DR, Jansen M, Broerse JEW, Wright P, Mayxay M. Improvement of Quality of Antenatal Care (ANC) Service Provision at the Public Health Facilities in Lao PDR: Perspective and Experiences of Supply and Demand Sides. BMC Pregnancy Childbirth. 2019;19(255):1–13. 31331276 10.1186/s12884-019-2345-0 PMC6647136

[17] Phommachanh S, Essink DR, Wright EP, Broerse JEW, Mayxay M. Do health care providers give sufficient information and good counseling during ante-natal care in Lao PDR?: an observational study. BMC Pregnancy Childbirth. 2019;19(449):1–12. 10.1186/s12913-019-4258-z 31272432 PMC6611023

[18] Ye Y, Yoshida Y, Harun-Or-Rashid M, Sakamoto J. Factors affecting the utilization of antenatal care services among women in Kham District, Xiengkhouang province, Lao PDR. Nagoya J Med Sci. 2010;72(1–2):23–33. 20229700 PMC11254371

[19] Manithip C, Sihavong A, Edin K, Wahlstrom R, Wessel H. Factors Associated with Antenatal Care Utilization Among Rural Women in Lao People ‘ s Democratic Republic. 2011;1356–62. 10.1007/s10995-010-0671-y 20827503

[20] Phathammavong O, Ali M, Souksavat S, Chounramany K, Kuroiwa C. Antenatal care among ethnic populations in Louang Namtha province, Lao PDR. Southeast Asian J Trop Med Public Health. 2010;41(3):705–16. 20578561

[21] Lao Statistics Bureau. Lao Social Indicator Survey II 2017, Survey Findings Report. Vientiane, Lao PDR; 2018.

[22] Barennes H, Simmala C, Odermatt P, Thaybouavone T, Vallee J, Martinez-Ussel B, et al. Postpartum traditions and nutrition practices among urban Lao women and their infants in Vientiane, Lao PDR. Eur J Clin Nutr. 2009;63(3):323–31. 10.1038/sj.ejcn.1602928 18000519 PMC3435433

[23] Phommachanh S, Essink DR, Wright EP, Broerse JEW, Mayxay M. Overt versus covert observations on health care providers’ care and communication during antenatal care visits in Lao PDR. J Glob Heal Sci. 2019;1(1):1–12.

[24] MOH. National Strategy and Action Plan for Integrated Services on Reproductive, Maternal, Newborn, and Child Health 2016–2025. 2015. 48 p.

[25] Mobley SC, Thomas SD, Sutherland DE, Hudgins J, Ange BL, Johnson MH. Maternal health literacy progression among rural perinatal women. Matern Child Health J. 2014;18(8):1881–92. 10.1007/s10995-014-1432-0 24469358 PMC4164840

[26] Calixte RE, Cnaan A. Participation in Social Welfare Programs. 2015;18(5):1176–89. 10.1007/s10995-013-1348-0 PMC3938979 23990157

[27] Su Beom Kim Eun Kyu Shine KTJ. Associations between maternal health literacy and prenatal care and pregnancy outcome. Iran J Nurs Midwifery Res Autumn 2007 [Internet]. 2010;4(1):1–7. Available from: http://kiss.kstudy.com/journal/thesis_name.asp?tname=kiss2002&key=3183676.

[28] De Souza RP, Foster PG, Sallum MAM, Coimbra TLM, Maeda AY, Silveira VR, et al. Exploring the Relationship between Maternal Health Literacy, Parenting Self-Efficacy, and Early Parenting Practices among Low-Income Mothers with Infants. J Med Virol. 2010;82(1):175–85. 10.1002/jmv.21606 19950229

[29] WHO. Pregnancy, Childbirth, Postpartum and Newborn Care: A guide for essential practice-3rd ed. 2015. 26561684

[30] Runk L, Durham J, Vongxay V, Sychareun V. Measuring health literacy in university students in Vientiane, Lao PDR. Health Promot Int. 2017;32(2):360–8. 10.1093/heapro/daw087 28011659

[31] Viengnakhone V., Alberts F., Thongmixay S., Thongsombath M., Broerse J., Sychareun V. EDR. Sexual and reproductive health Literacy of school adolescents in Lao PDR. Unpublished manuscript. 2017;1–15. Available from: 10.1371/journal.pone.0209675. PMC6334956 30650100

[32] Wright DB, Measelle J, Fong M, Einstein S, Niles J, Mobasser A, et al. Malnutrition in Lao PDR: Does maternal health knowledge buffer the negative effects of environmental risk factors on child stunting? Ann Glob Heal. 2016;82(3):572.

[33] Charoghchian Khorasani E, Peyman N, Esmaily H. Measuring Maternal Health Literacy in Pregnant Women Referred to the Healthcare Centers of Mashhad, Iran, in 2015. J Midwifery Reprod Heal. 2018;6(1):1157–62.

[34] Sørensen K, Broucke S Van Den, Fullam J, Doyle G, Pelikan J. Health literacy and public health: A systematic review and integration of definitions and models. BMC Public Health. 2012;12(1):80. 10.1186/1471-2458-12-80 22276600 PMC3292515

[35] DeWalt D a., Callahan LF, Hawk VH, Broucksou K a., Hink A, Rudd R, et al. Health Literacy Universal Precautions Toolkit. 2010;1–219. 10.1016/j.outlook.2010.12.002 PMC5091930 21402204

[36] Pelikan JM, Röthlin F, Ganahl K. Comparative Report On Health Literacy in Eight EU Member States. Online Publ. 2012;89.

[37] Journal CH. Maternal Health Literacy and Late Initiation of Immunizations Among an Inner-City Birth Cohort. 2011;15(3):386–94. 10.1007/s10995-010-0580-0 PMC6733024 20180003

[38] Pierre-louis JN, Sanjur D, Nesheim MC, Bowman DD, Mohammed HO. Maternal income-generating activities, child care, and child nutrition in Mali. 2014;28(1):67–75. 10.1177/156482650702800108 17718014

[39] Zerfu TA, Umeta M, Baye K. Dietary habits, food taboos, and perceptions towards weight gain during pregnancy in Arsi, rural central Ethiopia: a qualitative cross-sectional study. J Health Popul Nutr [Internet]. 2016;35(1):22. Available from: 10.1186/s41043-016-0059-8 27456151 PMC5025964

[40] Smedberg J, Lupattelli A, Mårdby AC, Nordeng H. Characteristics of women who continue smoking during pregnancy: A cross-sectional study of pregnant women and new mothers in 15 European countries. BMC Pregnancy Childbirth. 2014;14(1):1–16. 10.1186/1471-2393-14-213 24964728 PMC4080751

[41] Duong T V, Aringazina A, Baisunova G, Pham T V, Pham KM, Truong TQ, et al. Measuring health literacy in Asia: Validation of the HLS-EU-Q47 survey tool in six Asian countries. 2017;27:80–6. 10.1016/j.je.2016.09.005 PMC5328731 28142016

[42] Ganahl K, Slonska Z, Pelikan M, Ro F, Sørensen K. Health literacy in Europe: comparative results of the European health literacy survey (HLS-EU). 2015;25(6):1053–8. 10.1093/eurpub/ckv043 PMC4668324 25843827

[43] Renkert S, Nutbeam DON. Opportunities to improve maternal health literacy through antenatal education: an exploratory study. Health Promot Int. 2006;16(4):381–8. 10.1093/heapro/16.4.381 11733456

[44] Govindasamy P. Maternal Education and the Utilization of Maternal and Child Health Services in India. 1997;(5).

[45] Ede C, Chen X, Lin M, Chen YY. Exploring the Relationship between Maternal Health Literacy, Parenting Self-Efficacy, and Early Parenting Practices among Low-Income Mothers with Infants. HHS Public Access. 2018;5(5):395–404. 10.1353/hpu.2018.0106 PMC6320680 30449757

[46] Anderson M, Luangxay K, Sisouk K, Vorlasan L, Soumphonphakdy B, Sengmouang V, et al. Epidemiology of bacteremia in young hospitalized infants in vientiane, laos, 2000–2011. J Trop Pediatr. 2014;60(1):10–6. 10.1093/tropej/fmt064 23902672 PMC8210834

[47] Barennes H, Sengkhamyong K, René JP, Phimmasane M. Beriberi (Thiamine Deficiency) and High Infant Mortality in Northern Laos. PLoS Negl Trop Dis. 2015;9(3):1–16. 10.1371/journal.pntd.0003581 PMC4363666 25781926

[48] Soukaloun D, Lee SJ, Chamberlain K, Taylor AM, Mayxay M, Sisouk K, et al. Erythrocyte transketolase activity, markers of cardiac dysfunction and the diagnosis of infantile beriberi. PLoS Negl Trop Dis. 2011;5(2). 10.1371/journal.pntd.0000971 PMC3043003 21364976

[49] Khounnorath S, Chamberlain K, Taylor AM, Soukaloun D, Mayxay M, Lee SJ, et al. Clinically unapparent infantile thiamin deficiency in Vientiane, Laos. PLoS Negl Trop Dis. 2011;5(2):19–21. 10.1371/journal.pntd.0000969 21423705 PMC3050987

[50] Mayxay M, Taylor AM, Khanthavong M, Keola S, Pongvongsa T, Phompida S, et al. Thiamin deficiency and uncomplicated falciparum malaria in Laos. Trop Med Int Heal. 2007;12(3):363–9. 10.1111/j.1365-3156.2006.01804.x 17313507 PMC7611089

[51] Chen X, Zhao D, Mao X, Xia Y, Baker PN, Zhang H. Maternal dietary patterns and pregnancy outcome. Nutrients. 2016;8(6):1–26. 10.3390/nu8060351 27338455 PMC4924192

[52] Scheerhagen M, Van Stel HF, Birnie E, Franx A, Bonsel GJ. Measuring client experiences in maternity care under change: Development of a questionnaire based on the WHO responsiveness model. PLoS One. 2015;10(2):1–19. 10.1371/journal.pone.0117031 25671310 PMC4324965

